# Pirfenidone ameliorates pulmonary arterial pressure and neointimal remodeling in experimental pulmonary arterial hypertension by suppressing NLRP3 inflammasome activation

**DOI:** 10.1002/pul2.12101

**Published:** 2022-07-01

**Authors:** Emmanouil Mavrogiannis, Quint A. J. Hagdorn, Venetia Bazioti, Johannes M. Douwes, Diederik E. Van Der Feen, Silke U. Oberdorf‐Maass, Marit Westerterp, Rolf M. F. Berger

**Affiliations:** ^1^ Department of Pediatric Cardiology, Center For Congenital Heart Diseases, Beatrix Children's Hospital, University Medical Center Groningen University of Groningen Groningen The Netherlands; ^2^ Department of Pediatrics, Beatrix Children's Hospital, University Medical Center Groningen University of Groningen Groningen The Netherlands; ^3^ Department of Experimental Cardiology, University Medical Center Groningen University of Groningen Groningen The Netherlands

**Keywords:** caspase‐1, IL‐18, IL‐1β, vascular remodeling

## Abstract

Pulmonary arterial hypertension (PAH) is a fatal disease characterized by increased pulmonary arterial pressure, inflammation, and neointimal remodeling of pulmonary arterioles. Serum levels of interleukin (IL)‐1β and IL‐18 are elevated in PAH patients and may enhance proinflammatory neointimal remodeling. NLRP3 inflammasome activation induces cleavage of the cytokines IL‐1β and IL‐18, required for their secretion. Pirfenidone (PFD), an antifibrotic and anti‐inflammatory drug, has been suggested to inhibit NLRP3 inflammasome activation. We hypothesized that PFD delays the progression of PAH by suppressing NLRP3 inflammasome activation. We assessed the effects of PFD treatment in a rat model for neointimal PAH induced by monocrotaline and aortocaval shunt using echocardiographic, hemodynamic, and vascular remodeling parameters. We measured inflammasome activation by NLRP3 immunostaining, Western blots for caspase‐1, IL‐1β, and IL‐18 cleavage, and macrophage IL‐1β secretion. PFD treatment ameliorated pulmonary arterial pressure, pulmonary vascular resistance, and pulmonary vascular remodeling in PAH rats. In PAH rats, immunostaining of NLRP3 in pulmonary arterioles and caspase‐1, IL‐1β, and IL‐18 cleavage in lung homogenates were increased compared to controls, reflecting NLRP3 inflammasome activation in vivo. PFD decreased IL‐1β and IL‐18 cleavage, as well as macrophage IL‐1β secretion in vitro. Our studies show that PFD ameliorates pulmonary hemodynamics and vascular remodeling in experimental PAH. Although PFD did not affect all NLRP3 inflammasome parameters, it decreased IL‐1β and IL‐18 cleavage, the products of NLRP3 inflammasome activation that are key to its downstream effects. Our findings thus suggest a therapeutic benefit of PFD in PAH via suppression of NLRP3 inflammasome activation.

## INTRODUCTION

Pulmonary arterial hypertension (PAH) is a rare, progressive, and currently incurable disease, characterized by inflammation and remodeling of distal pulmonary arterioles resulting in increased pulmonary arterial pressure (mPAP) and right ventricular (RV) afterload. This eventually leads to right heart failure and death.^1^ The process of pulmonary vascular remodeling, including neointimal lesions, is orchestrated by endothelial inflammation, changes in the extracellular matrix, and chemoattraction of leukocytes.^2^, ^3^, ^4^

Although the precise mechanisms remain unclear, inflammation has been recognized as a major contributor to the pathogenesis of the pulmonary vascular remodeling process in PAH.^2^, ^3^, ^4^ In patients with PAH, plasma levels of interleukin‐1β (IL‐1β) and IL‐18, major regulators of inflammation, are elevated.^5^, ^6^ The secretion of these two proinflammatory cytokines is regulated by inflammasomes, intracellular multimeric protein complexes. The hallmark of inflammasome activation is the cleavage of its key enzyme caspase‐1. Cleaved, active caspase‐1 then cleaves the proforms of IL‐1β and IL‐18, which is required for their secretion.^7^, ^8^

Specifically, the NLRP3 inflammasome, consisting of the NLRP3 sensor, the apoptosis‐associated speck‐like protein (ASC), and caspase‐1, has recently been proposed as a major driver of cardiovascular diseases (CVDs).^9^ The NLRP3 inflammasome requires two signals for activation: a priming signal that activates the nuclear factor (NF)‐κB, which enhances the messenger RNA (mRNA) transcription of pro‐IL‐1β, and the NLRP3 inflammasome components NLRP3, ASC, and caspase‐1, and a triggering signal that leads to the cleavage of caspase‐1. The NLRP3 inflammasome has originally been identified in macrophages.^6^ Later studies also showed high expression in endothelial cells.^10^, ^11^ Moreover, disturbed blood flow, a well‐recognized trigger for PAH in humans,^12^, ^13^ has been shown to activate the endothelial NLRP3 inflammasome in vitro, by functioning both as a priming and activation signal.^10^, ^11^ Recent data from animal studies suggest a role for the NLRP3 inflammasome in the progression of pulmonary hypertension (PH), but its exact contribution is still poorly defined.^14^, ^15^

Current therapeutic options have improved the survival of patients with PAH but are certainly not curative and the prognosis is still poor.^1^ A cure that can reverse the adverse pulmonary vascular remodeling in PAH is yet to be introduced in the clinic. Pirfenidone (5‐methyl‐1‐phenyl‐2‐[1H]‐pyridone, or PFD) is a synthetic pyridone derivative with antifibrotic, anti‐inflammatory, and antioxidative properties and is clinically used for the treatment of idiopathic pulmonary fibrosis, with a good safety profile.^16^ Of interest, PFD has been suggested to suppress NLRP3 inflammasome activation in animal models of pulmonary and cardiac fibrosis.^17^, ^18^, ^19^

In this study, we investigated whether the NLRP3 inflammasome is activated in a rat model of flow‐associated neointimal PAH, induced by monocrotaline (MCT), and aortocaval shunt and whether early PFD treatment affects NLRP3 inflammasome activation, pulmonary hemodynamics, and vascular remodeling.

## METHODS

### Animal experiments

Twenty‐six male young adult Wistar rats were randomly divided into three groups: a control group (control; *N* = 6), a group with induced PAH (PAH; *N* = 10), and a group with induced PAH that received PFD treatment (PAH+PFD; *N *= 10). For induction of PAH, the animals received 60 mg/kg pyrrolizidine alkaloid MCT (Sigma Chemical Co.) subcutaneously and 7 days later underwent aortocaval shunt surgery.^20^ The control animals received saline injection instead of MCT and underwent sham surgery. For the PAH+PFD group, 0.4% PFD was mixed in the chow from day 7 onwards.^17^, ^21^ On day 28, the animals were anesthetized, underwent echocardiography and closed‐chest right heart catheterization, and were subsequently sacrificed by cardiac exsanguination, and lungs were collected.^21^ One rat from the PAH+PFD group died shortly after surgery, attributed to a procedural complication. Another rat from the PAH+PFD group was found dead in its cage on day 25, and findings from the autopsy suggested right heart failure.

### Hemodynamic evaluation

Echocardiography and right heart catheterization were performed as previously described.^20^ Echocardiographic measurements included tricuspid annular plane systolic excursion (TAPSE) to evaluate the RV function, and pulmonary artery acceleration time (PAAT reflecting pulmonary vascular resistance, PVR). Invasive hemodynamic measurements included systolic RV pressure, mean pulmonary artery pressure, and pulmonary arterial wedge pressure. PVR was calculated. Indexing of cardiovascular parameters was performed using tibia length, as described previously.^22^

### Histology and immunohistochemistry

Formalin‐fixed paraffin‐embedded lungs were cut into 4 µm sections. Pulmonary sections were stained with hematoxylin‐eosin and elastin stain (HT25A‐1KT; Sigma‐Aldrich) for qualitative and quantitative morphometric analysis of pulmonary arterioles and scanned in ×40 magnification with a large‐scale digital scanner (Hamamatsu Photonics). After blinding, each lung tissue scan was divided into four quadrants. In every quadrant, the first five intra‐acinar arterioles (with diameter <50 µm) that fulfilled the selection criteria as previously described were selected and used for further analysis.^20^ External and internal arteriolar areas, medial and neointimal thicknesses were measured using FIJI‐ImageJ (NIH Image) and the arteriolar occlusion score was calculated, all according to previously published protocols.^20^ To assess fibrosis, Masson's trichrome staining was performed on 4 µm lung sections and scanned by a large‐scale digital scanner (Hamamatsu Photonics). Positive pixel count with the software aperio image scope (version 12.4.0.5043; Leicabiosystems) was used for quantification. Specifically, 20 intra‐acinar arterioles, five per lung quadrant, were randomly chosen and analyzed from which a mean was drawn and used for the statistical analysis. For the NLRP3 immunohistochemical staining on lung sections, we used NLRP3 antibodies (ab214185; Abcam; 1:100), while using goat anti‐rabbit horseradish peroxidase(HRP) as a secondary antibody (P044801; Dako). 3,3′‐Diaminobenzidine was used to detect NLRP3 staining and the sections were counter‐stained with hematoxylin. For CD68^+^ cell staining, we used anti‐CD68 (1:100 in phosphate‐buffered saline [PBS]/1% bovine serum albumin) (MCA341R AbD serotec GA) as primary and rabbit‐anti‐mouse immunoglobulins/HRP (P0260; Dako) as the secondary antibody. Aminoethyl carbazole chromogen was used to detect the CD68^+^ cells and the sections were counter‐stained with hematoxylin. On the scans by Aperio CS2 and Hamamatsu slide scanner, 20 intra‐acinar arterioles, five per quartile of the lung section were randomly selected, and NLRP3 positive cells in the intima and media were counted. The number of positive cells per 10 arterioles was used for statistical analysis. For the immunofluorescent stainings, we used as primary antibodies anti‐NLRP3 (NLRP3/NALP3 antibody [NBP2‐12446]: Novus Biologicals), anti‐CD68 (MCA341, ED1 clone; Bio‐Rad Laboratories), and anti‐α‐smooth muscle actin (α‐SMA) (67735‐1‐Ig; Proteintech). As secondary antibodies, we used immunoglobulin G (IgG) goat‐anti‐mouse secondary fluor 647 (ab150115; Abcam) or goat anti‐rabbit IgG (H + L) highly cross‐adsorbed secondary antibody, Alexa Fluor 488 (#A‐11034; Thermo Fisher Scientific). The sections were counterstained with 4′6‐diamidino‐2‐phenylindole (P36941; Invitrogen) and photos were made by Zeiss 410 inverted laser scan microscope (Leica Microsystems).

### Immunoblotting

Lung tissues were snap‐frozen and protein was isolated. For caspase‐1 immunoblotting, semi‐dry membrane transfer was performed. Anti‐caspase‐1 (14F468) 1:200 (sc‐56036; Santa Cruz Biotechnology Inc.) and anti‐GAPDH 1:30,000 were used as primary antibodies, and rabbit anti‐mouse HRP 1:2500 in 5% fat‐free milk in TBST (P0260; Dako) as the secondary antibody. For IL‐1β and IL‐18 immunoblotting, wet membrane transfer was performed. Anti‐IL‐1β (1:1000) (IL‐1β IL‐F2 NBP1‐42767; Novus Biologicals) and anti‐IL‐18 (1 µg/ml) (IL‐18/IL‐1F4, AF521; R&D Systems) were used as primary antibodies and anti‐rabbit IgG HRP (#1706515; Bio‐Rad Laboratories Inc.) or anti‐goat IgG HRP‐linked antibody (1:2000) (sc‐2020; Santa Cruz Biotechnology Inc.) as secondary antibodies, respectively. All antibodies were diluted in 2.5% fat‐free milk in TBS‐T. FIJI‐ImageJ (NIH) was used for the quantification of the immunoblots. The cleavage ratio for caspase‐1, IL‐1β, and IL‐18 was calculated as cleaved caspase‐1 (p20) divided by pro‐caspase‐1 (p45), or cleaved IL‐1β (p15) divided by pro‐IL‐1β (p34), or cleaved IL‐18 (p18) divided by pro‐IL‐18 (p24).

### Inflammasome assays in bone marrow‐derived macrophages

Tibias and femurs were isolated from C57BL6 mice and flushed with PBS to obtain bone marrow (BM) cells. BM cells were cultured in Dulbecco's modified Eagle's medium supplemented with 10% fetal bovine serum, 1% pen–strep, and 20% L929‐cell‐conditioned medium for 7 days to induce complete differentiation into bone marrow‐derived macrophages (BMDMs), as previously described.^23^ Then, BMDMs were treated o/n with or without PFD (500 µg/ml), and subsequently stimulated with lipopolysaccharide (LPS) (100 ng/ml) for 4 h. Cells were lysed, RNA was extracted using the Qiagen RNeasy Kit (74004; QIAGEN) and complementary DNA (cDNA) was synthesized using the Transcriptor Universal cDNA Master Kit (Roche). Subsequently, mRNA expression of NLRP3 (forward [F]: 5′‐ATTACCCGCCCGAGAAAGG‐3′; reverse [R]: 5′‐TCGCAGCAAAGATCCACACAG‐3′), IL‐1β (F: 5′‐TGCAGCTGGAGAGTGTGG‐3′; R: 5′‐TGCTTGTGAGGTGCTGATG‐3′), and IL‐18 (F: 5′‐ GACTCTTGCGTCAACTTCAAGG‐3′; R: 5′‐CAGGCTGTCTTTTGTCAACGA‐3′) was assessed by quantitative polymerase chain reaction (PCR) using QuantStudio 7 Flex Real‐Time PCR System (Applied Biosystems). Initial differences in RNA quantity were corrected by using the housekeeping genes GAPDH (F: 5′‐ATTGTCAGCAATGCATCCTG‐3′; R: 5′‐ ATGGACTGTGGTCATGAGCC‐3′), m36B4 (F: 5′‐GGACCCGAGAAGACCTCCTT‐3′; R: 5′‐GCACATCACTCAGAATTTCAATGG‐3′), and cyclophilin B (F: 5′‐ATGGCACAGGAGGAAAGAGC‐3′; R: 5′‐ ATGATCACATCCTTCAGGGG‐3′).

Alternatively, after LPS stimulation, BMDMs were subsequently treated with nigericin (20 µM) for 30 min. IL‐1β secretion into the medium was assessed using an enzyme‐linked immunosorbent assay (ELISA; IL‐1β/IL‐F2, MLB00C; R&D Systems). Cell protein was measured by bicinchoninic acid assay (23235; Thermo Fisher Scientific), and IL‐1β secretion was corrected for cell protein.

### Statistical analysis

For comparison of the three groups, two‐sided one‐way was performed with Holm–Sidak's post hoc correction for multiple comparisons in GraphPad Prism 8 (GraphPad). Effects of PFD on the progression of PAH were the pulmonary hemodynamic variables mPAP and PVR and the histopathologic variables indicative of vascular remodeling including vascular occlusion score, and perivascular fibrosis content. Outcome measures for assessing inflammasome activation were caspase‐1, IL‐1β, and IL‐18 cleavage, and NLRP3 immunostaining. SPSS Statistics version 26 (IBM) was used for the linear regressions and the scatter plots of all the animals to test whether perivascular fibrosis as well as inflammasome priming or activation correlated with markers of pulmonary vascular disease severity. For the comparison of two groups of the BMDM experiment, we performed an unpaired, two‐tailed *T*‐test in GraphPad Prism 8 (GraphPad). *p* ≤ 0.05 were considered significant. Results are shown as means ± standard deviation.

## RESULTS

### PFD ameliorates experimental flow‐associated neointimal PAH

**FIGURE 1:**
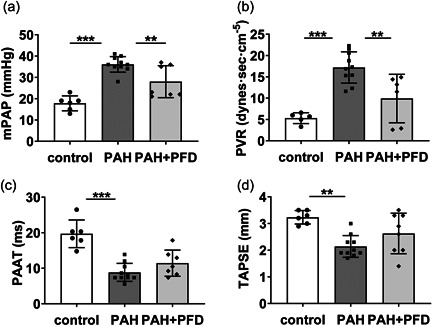
Effects of pirfenidone (PFD) on hemodynamics in the monocrotaline and aortocaval shunt rat model for PAH: PAH was induced in rats by intraperitoneal injection of 1 ml (60 mg/ml) monocrotaline followed by aortocaval shunt surgery 7 days later. Controls received injections with saline and underwent sham surgery. PAH animals were randomly divided into two groups: the PAH and PAH+PFD groups. The PAH group received a chow diet. The PAH+PFD group received a chow diet supplemented with 0.4% PFD. Hemodynamics were assessed 20 days after aortocaval shunt surgery. (a) mPAP: mean pulmonary arterial pressure; (b) PVR: pulmonary vascular resistance; (c) PAAT: pulmonary artery acceleration time; and (d) TAPSE: tricuspid annular plane systolic excursion. *p < 0.05; **p < 0.01; ***p < 0.001 by two‐sided one‐way ANOVA with Holm–Sidak's post hoc correction. ANOVA, analysis of variance; PAH, pulmonary arterial hypertension.

We first investigated hemodynamic and histopathologic parameters in the PAH group compared to the control group and subsequently the effects of PFD. Compared to controls, animals in the PAH group showed increased mPAP and PVR measured by cardiac catheterization on Day 28 and decreased TAPSE and PAAT measured by echocardiography (Figure 1a–d). PFD decreased mPAP and PVR on Day 28 in PAH rats (Figure 1a,b), but did not significantly affect echocardiographic measurements of TAPSE and PAAT (Figure 1c,d).

**FIGURE 2:**
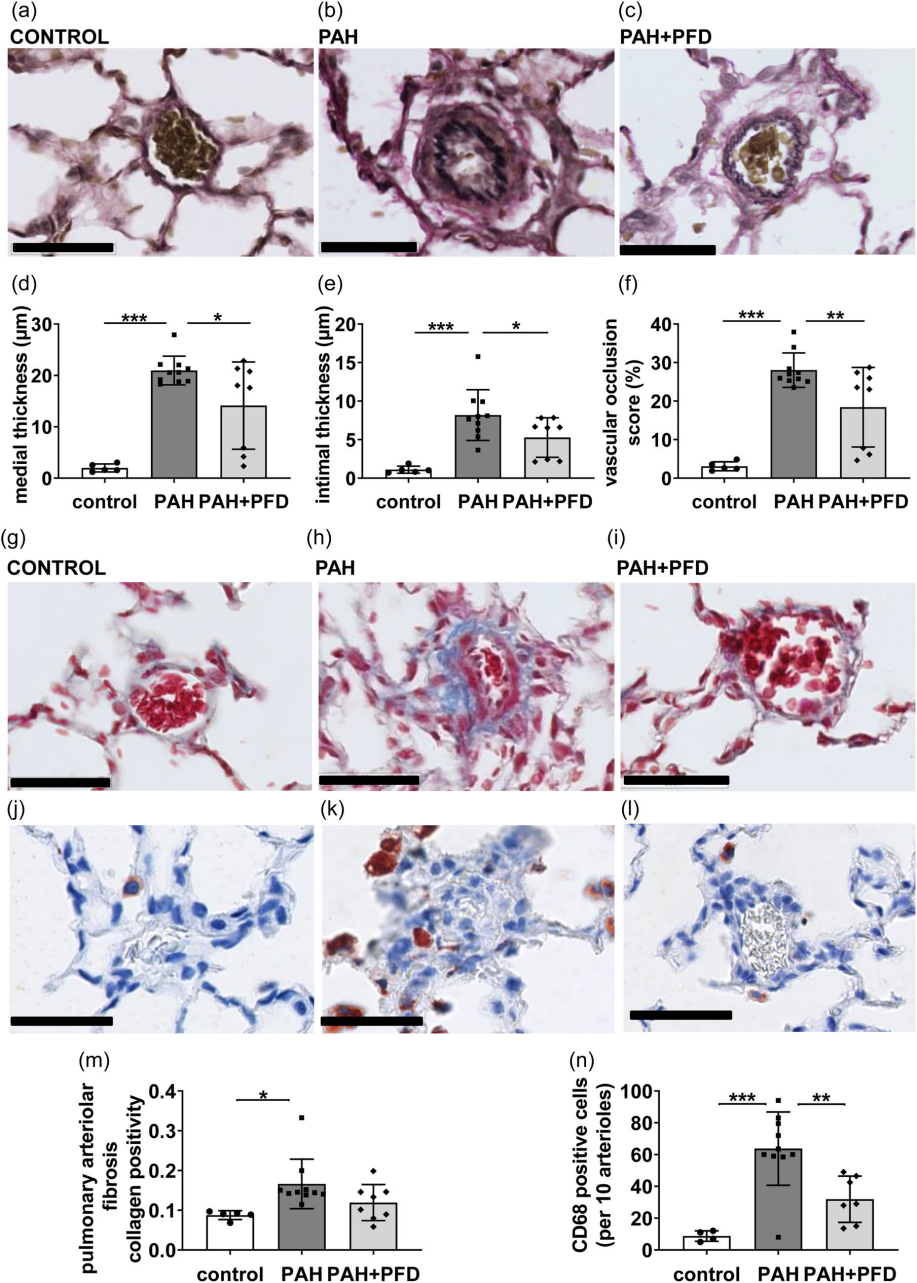
Effects of pirfenidone (PFD) on pulmonary vascular remodeling: Lungs were collected 20 days after aortocaval shunt surgery, embedded in paraffin, sectioned (4 µm), and stained. (a–c) Representative pictures of elastin staining of pulmonary arterioles; (d) pulmonary arteriolar medial thickness; (e) pulmonary arteriolar intimal thickness; (f) pulmonary arteriolar vascular occlusion score; (g–i) representative pictures of Masson's trichrome staining of pulmonary arterioles and collagen fibers stain blue; (j–l) representative pictures of CD68 positive cells (in red) close to pulmonary arterioles; (m) pulmonary arteriolar fibrosis; and (n) CD68 positive cells counted per 10 pulmonary arterioles; scale bar = 50 µm; *p < 0.05; **p < 0.01; ***p < 0.001 by two‐sided one‐way ANOVA with Holm–Sidak's post hoc correction. ANOVA, analysis of variance; PAH, pulmonary arterial hypertension.

Histomorphometry revealed that in comparison to the control group, PAH rats had increased intimal and medial thickness of the pulmonary arteriolar walls and increased arteriolar occlusion score (Figure 2a–f). PFD decreased the thickness of both intimal and medial layers of the pulmonary arteriolar walls (Figure 2d,e) and arteriolar occlusion score in PAH rats (Figure 2f), implying less adverse pulmonary vascular remodeling. PAH rats showed an increased number of perivascular CD68+ macrophages and increased perivascular fibrosis compared to controls (Figure 2g–n). PFD treatment decreased the number of perivascular CD68^+^ macrophages (Figure 2n), whereas it did not significantly affect perivascular fibrosis (Figure 2m). For the perivascular fibrosis, we found a positive significant correlation with the disease severity markers mPAP and occlusion score (*R* ^2^ = 0.226, *p* = 0.025 and *R* ^2^ = 0.471, *p* < 0.001, respectively).

These results indicate that PFD treatment beneficially affected pulmonary hemodynamics and pulmonary vascular remodeling in this rat model for neointimal PAH, whereas an effect on cardiac adaptation could not be demonstrated at day 28.

In the treated animals, we observed a heterogeneous, almost dichotomous response to PFD treatment, with respect to pulmonary hemodynamics, vascular remodeling, and cardiac adaptation (Figures 1a–d and 2a–n). Linear regression revealed that RV function, expressed as TAPSE, correlated significantly with mPAP (*β* = −0.702, *p* < 0.001) and with the pulmonary arteriolar occlusion score (*β* = −0.813, *p* < 0.001). This implies that the animals with a more advanced state of vascular remodeling and higher PAP showed more compromised RV function.

### The NLRP3 inflammasome is activated in experimental flow‐associated neointimal PAH and ameliorated by PFD

**FIGURE 3:**
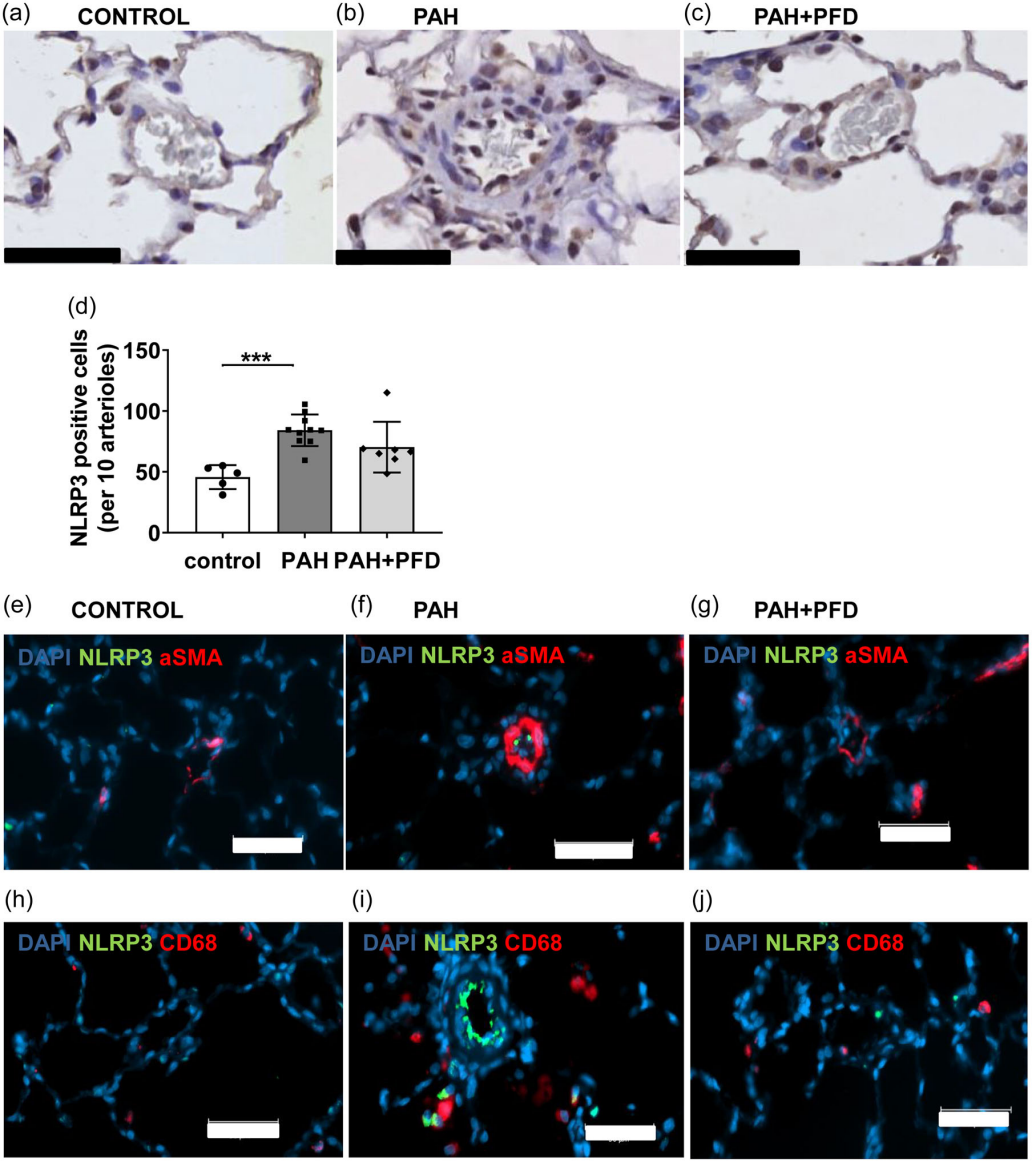
PAH induces NLRP3 expression in the distal pulmonary bed: Paraffin sections from lungs were stained for NLRP3. (a–c) Representative pictures of pulmonary arterioles stained for NLRP3 (dark brown); (d) number of NLRP3 positive cells per 10 pulmonary arterioles; (e–g) representative arterioles of double stainings of NLRP3 (green) and α‐smooth muscle actin (α‐SMA) in red; and (h–j) representative arterioles of double stainings of NLRP3 in green and CD‐68 in red; scale bar = 50 µm; ***p < 0.001 by two‐sided one‐way ANOVA with Holm–Sidak's post hoc correction. ANOVA, analysis of variance; DAPI, 4′,6‐diamidino‐2‐phenylindole; PAH, pulmonary arterial hypertension; PFD, pirfenidone.

**FIGURE 4:**
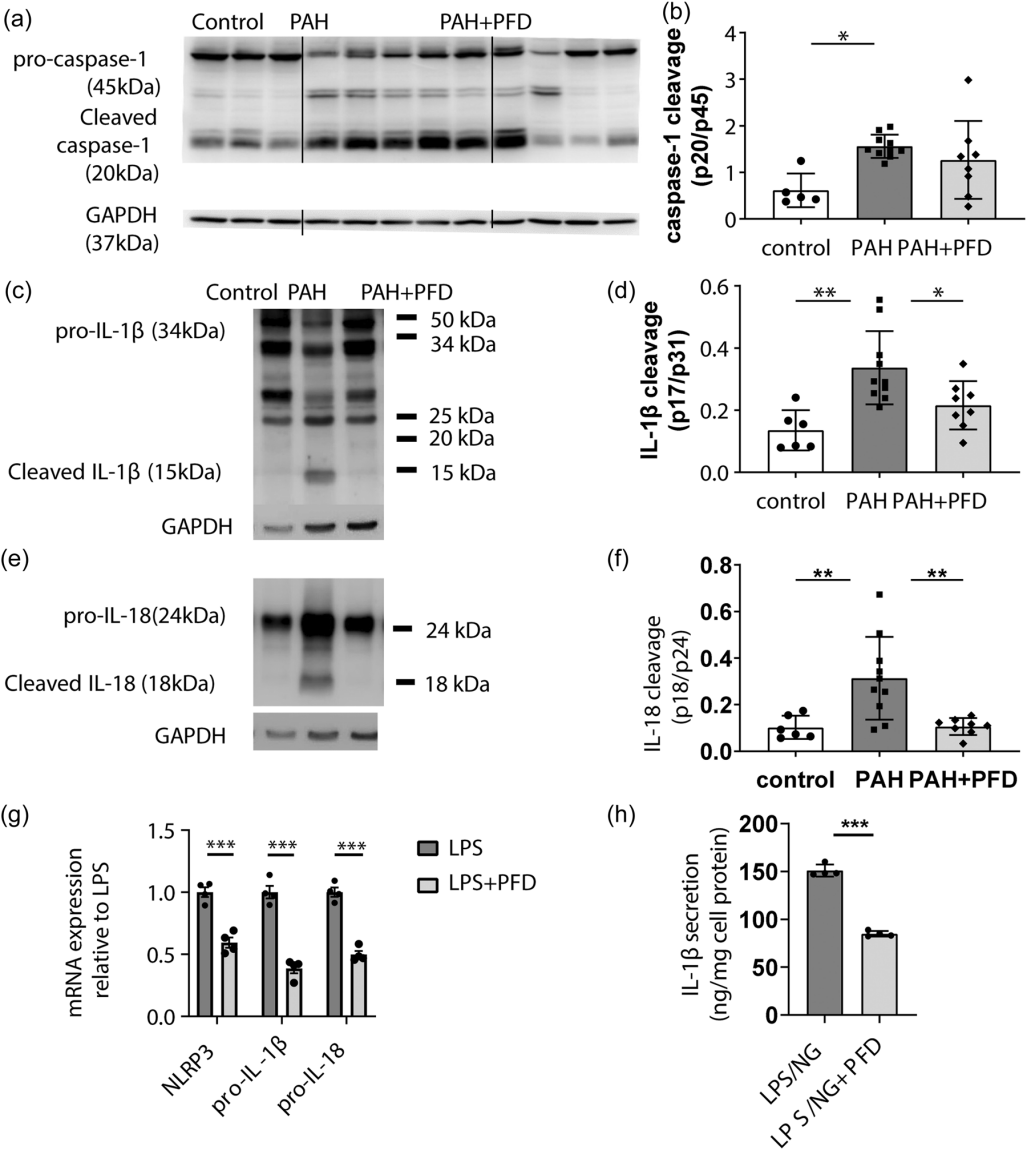
Effects of pirfenidone (PFD) on NLRP3 inflammasome activation in PAH lungs and in macrophages in vitro: Lung tissue was isolated from the rats as described in Figures 1, 2, 3, homogenized and cleavage of caspase‐1 (a, b), IL‐1β (c, d), and IL‐18 (e, f) were assessed by Western blot. Representative pictures are shown (a, c, e), and quantified (b, d, f). (g, h) Bone marrow was isolated from mice and differentiated into bone marrow‐derived macrophages (BMDMs) in DMEM supplemented with a 20% l‐cell conditioned medium for 7 days. Subsequently, BMDMs were treated with or without PFD (500 µg/ml) o/n, and subsequently with lipopolysaccharide (LPS) (100 ng/ml) for 4 h. (g) Cells were lysed, RNA was isolated, and the expression of NLRP3, pro‐IL‐1β, and pro‐IL‐18 mRNA was measured by qPCR and corrected for 36B4, cyclophilin B, and GAPDH (housekeeping genes). (h) Cells were subsequently treated with nigericin (NG) (20 µM) for 30 min and IL‐1β secretion into the medium was assessed by ELISA and corrected for cell protein; *p < 0.05; **p < 0.01; ***p < 0.001 by two‐sided one‐way ANOVA with Holm–Sidak's post hoc correction. ANOVA, analysis of variance; ELISA, enzyme‐linked immunosorbent assay; IL‐1β, interleukin‐1β; mRNA, messenger RNA; PAH, pulmonary arterial hypertension; qPCR, quantitative polymerase chain reaction.

We then investigated whether PAH in this model was associated with an increase in the activation of NLRP3 inflammasomes. Immunohistochemistry revealed an increase in NLRP3 positive cells in pulmonary arterioles of PAH animals when compared to the control group (Figure 3a–d), suggesting increased NLRP3 inflammasome priming. Using double immunostainings, we investigated which cell types were positive for NLRP3. We found no colocalization of NLRP3 and α‐SMA staining, suggesting that NLRP3 expression in SMCs was not affected in PAH (Figure 3e–g). However, NLRP3 staining did colocalize with some perivascular CD68^+^ cells in PAH (Figure 3h–j). Moreover, cells located at the luminal border of the vessel wall, likely being endothelial cells, were highly NLRP3 positive in the PAH condition but not in control or PAH+PFD (Figure 3e–j). These data are suggestive of NLRP3 inflammasome priming in endothelial cells and perivascular macrophages in PAH. We then assessed caspase‐1 cleavage, which reflects the ratio of cleaved caspase‐1 (p20) to its proform (p45) and is a hallmark of inflammasome activation, in the lung homogenates using Western blot (Figure 4a,b). Animals in the PAH group showed increased caspase‐1 cleavage compared to control animals. PFD showed a trend of decreased caspase‐1 cleavage in PAH rats, but this did not reach statistical significance (Figure 4a,b). However, NLRP3 positive cells and cleaved caspase‐1 correlated with the disease severity measured by mPAP and by occlusion score (NLRP3: *R* ^2^ = 0.483, *p* = 0.023 and *R* ^2^ = 0.776, *p* < 0.00, respectively; cleaved caspase‐1: *R* ^2^ = 0.514, *p* < 0.00, *R* ^2^ = 0.388, *p* = 0.004, respectively). Cleaved IL‐1β and IL‐18, products of NLRP3 inflammasome activation, were also significantly increased by twofold and threefold, respectively, in lung homogenate of PAH animals compared to control, which was reversed by PFD (35% and 65%, respectively) (Figure 4c–f). Together, these findings indicate increased inflammasome activation in advanced experimental flow‐associated PAH with reversal by PFD. Even though PFD did not reverse all parameters of NLRP3 inflammasome activation, it did decrease cleaved IL‐1β and IL‐18, which are key to the downstream effects of NLRP3 inflammasome activation.^7^, ^8^ Cleaved IL‐1β and IL‐18 are also elevated in the plasma of PAH patients.^5^, ^6^ These data suggest that PFD may exert beneficial effects in PAH by inhibiting NLRP3 inflammasome activation.

### PFD treatment suppresses NLRP3 inflammasome activation in macrophages

As the NLRP3 inflammasome was activated in perivascular macrophages in PAH, and PFD reversed several parameters of NLRP3 inflammasome activation, we investigated the effects of PFD on NLRP3 inflammasome activation in vitro in BMDMs. BMDMs stimulated with LPS showed upregulation of NLRP3, pro‐IL‐1β, and pro‐IL‐18 mRNA expression, which was reduced by PFD (Figure 4g), suggesting that PFD inhibits NLRP3 inflammasome priming. To investigate whether this resulted in a decrease in IL‐1β secretion, a product of NLRP3 inflammasome activation, we stimulated BMDMs with LPS and nigericin to achieve complete NLRP3 inflammasome activation. PFD reduced IL‐1β secretion into the medium (Figure 4h), indicating that the suppression of NLRP3 inflammasome priming by PFD results in a decrease in IL‐1β secretion.

## DISCUSSION

This study demonstrates that the NLRP3 inflammasome is activated in the well‐established PAH rat model for advanced, flow‐associated neointimal PAH induced by MCT and aortocaval shunt, which reflects PAH in humans. NLRP3 inflammasome activation is reflected by increased numbers of NLRP3 positive cells in pulmonary arterioles, increased cleavage of caspase‐1, and the inflammasome products IL‐1β and IL‐18 in the whole lung tissue. Although the inflammasome has been suggested to be activated in several PH models,^14^, ^15^, ^24^, ^25^, ^26^, ^27^ human translational relevance of these models is limited.

PFD treatment suppressed pulmonary vascular disease development in the used neointimal PAH rat model. While PFD did not affect the total number of NLRP3 positive cells in pulmonary arterioles nor caspase‐1 cleavage, it did inhibit IL‐1β and IL‐18 cleavage in lung tissue, indicating that PFD inhibited the production of the main NLRP3 inflammasome products that regulate its downstream effects. PFD decreased perivascular CD68^+^ macrophages. This may have been the consequence of decreased cleaved, active IL‐1β in the lung as active IL‐1β is a known promotor of monocyte/macrophage recruitment.^28^ Furthermore, IL‐1β induces medial hypertrophy and vascular remodeling by promoting SMC proliferation^29^, ^30^, ^31^ and fibrosis.^32^ We, therefore, conclude that the hemodynamic and vascular morphometric improvement by PFD in PAH may be downstream of decreased IL‐1β production mediated by the NLRP3 inflammasome. Mechanistic studies in macrophages in vitro showed that PFD reduces NLRP3 inflammasome priming, in line with previous observations.^18^, ^19^ This resulted in a decrease in IL‐1β secretion, substantiating inhibition of NLRP3 inflammasome activation by PFD. In addition, we cannot exclude that PFD may also have inhibited IL‐1β cleavage in PAH van caspase‐independent pathways such as those mediated by neutrophil elastase or proteinase‐3.^33^

In the past decade, studies on the pathobiology of PAH have revealed substantial evidence that inflammation plays a key role in PH pathogenesis and that pulmonary vascular remodeling may be driven by aberrant immune responses.^2^, ^3^, ^4^ However, current understanding of the inflammatory pathways involved in PH is still limited. Specifically, IL‐1β and IL‐18 plasma levels have been shown to be elevated in PAH patients.^5^, ^6^, ^14^, ^15^ The production of IL‐1β and IL‐18 is regulated by inflammasomes.^7^ The current study suggests that the increase in plasma IL‐1β and IL‐18 in PAH patients may be the consequence of pulmonary NLRP3 inflammasome activation. This will have implications for progressive human PAH, including PAH associated with congenital heart disease.^14^, ^15^, ^20^, ^24^, ^25^, ^26^, ^27^

The canakinumab anti‐inflammatory thrombosis outcomes (CANTOS) study has shown that antagonism of IL‐1β reduces recurrent myocardial infarction in patients with plasma C‐reactive protein (CRP) levels >2 mg/L, suggesting an important role for IL‐1β induced inflammation in CVD.^9^ A small‐scale pilot trial in adult PAH patients showed that IL‐1 receptor blockade by anakinra reduced plasma CRP within 15 days of treatment, yet this did not result in significant hemodynamic or echocardiographic changes.^34^ However, that study will need to be expanded, and the duration may need to be increased to conclusively assess whether inflammation‐induced via the IL‐1 receptor, and mediated by IL‐1β contributes to PAH in humans.

### Limitations

We found heterogenous hemodynamic and histopathologic responses in the PFD‐treated PAH rats. One of the reasons may be a difference in chow intake and thus PFD administration, as the appetite of the rats may have been affected by PAH. We did not monitor food intake or blood levels of PFD and thus cannot exclude heterogeneous PFD blood levels in the treated rats. However, oral administration of 0.4% PFD in the chow diet has been reported as a reliable method in various animal models^17^, ^21^, in future studies other ways of PFD administration or more tightly control of PFD concentration in blood may be considered.

In summary, our studies show that PFD ameliorates pulmonary hemodynamics and vascular remodeling in experimental PAH. Although PFD did not affect all NLRP3 inflammasome parameters, it decreased IL‐1β and IL‐18 cleavage, the products of NLRP3 inflammasome activation that are keys to its downstream effects. Our findings thus suggest that NLRP3 inflammasome activation could be a therapeutic target in PAH.

## AUTHOR CONTRIBUTIONS

Emmanouil Mavrogiannis participated in the study design, data acquisition, statistical analysis, and drafting of the manuscript. Quint A. J. Hagdorn participated in the study design, data acquisition, statistical analysis, and revising of the manuscript. Venetia Bazioti participated in the study design, data acquisition, statistical analysis, and revising of the manuscript. Johannes M. Douwes participated in the study design and revising of the manuscript. Diederik E. Van Der Feen participated in the study design and revising of the manuscript. Silke U. Oberdorf‐Maass participated in data acquisition and revising of the manuscript. Marit Westerterp participated in the study design, data interpretation, statistical analysis, and drafting of the manuscript. Rolf M. F. Berger participated in the study design, data interpretation, drafting, and revising of the manuscript.

## CONFLICT OF INTEREST

The University Medical Center Groningen contracts with Actelion, GSK, and Lilly for the steering committee and advisory board activities of Prof. Dr. Rolf M. F. Berger outside the content of this manuscript. The rest of the authors declare no conflict of interest.

## ETHICS STATEMENT

All animal studies were approved by the Institutional Animal Care and Use Committee from the University of Groningen under permit number AVD10500201512 and adhered to the guidelines set out in the 2010/63/EU directive, the 1964 Declaration of Helsinki, and its later amendments.

## Data Availability

Data are available on request from the authors.
